# Differential Response of Mouse Thymic Epithelial Cell Types to Ionizing Radiation-Induced DNA Damage

**DOI:** 10.3389/fimmu.2017.00418

**Published:** 2017-04-13

**Authors:** Irene Calvo-Asensio, Thomas Barthlott, Lilly von Muenchow, Noel F. Lowndes, Rhodri Ceredig

**Affiliations:** ^1^Regenerative Medicine Institute, School of Medicine, Nursing and Health Sciences, National University of Ireland, Galway, Ireland; ^2^Genome Stability Laboratory, Centre for Chromosome Biology, School of Natural Sciences, National University of Ireland, Galway, Ireland; ^3^Pediatric Immunology, Department of Biomedicine, University Children’s Hospital (UKBB) and University of Basel, Basel, Switzerland; ^4^Developmental and Molecular Immunology, Department of Biomedicine, University of Basel, Basel, Switzerland

**Keywords:** thymic epithelial cells, DNA damage response, ionizing radiation, hypoxia, bone marrow transplantation

## Abstract

Thymic epithelial cells (TECs) are the main components of the thymic stroma that support and control T-cell development. Preparative regimens using DNA-damaging agents, such as total body irradiation and/or chemotherapeutic drugs, that are necessary prior to bone marrow transplantation (BMT) have profound deleterious effects on the hematopoietic system, including the thymic stroma, which may be one of the main causes for the prolonged periods of T-cell deficiency and the inefficient T cell reconstitution that are common following BMT. The DNA damage response (DDR) is a complex signaling network that allows cells to respond to all sorts of genotoxic insults. Hypoxia is known to modulate the DDR and play a role affecting the survival capacity of different cell types. In this study, we have characterized in detail the DDR of cortical and medullary TEC lines and their response to ionizing radiation, as well as the effects of hypoxia on their DDR. Although both mTECs and cTECs display relatively high radio-resistance, mTEC cells have an increased survival capacity to ionizing radiation (IR)-induced DNA damage, and hypoxia specifically decreases the radio-resistance of mTECs by upregulating the expression of the pro-apoptotic factor Bim. Analysis of the expression of TEC functional factors by primary mouse TECs showed a marked decrease of highly important genes for TEC function and confirmed cTECs as the most affected cell type by IR. These findings have important implications for improving the outcomes of BMT and promoting successful T cell reconstitution.

## Introduction

The thymus is the main organ for T lymphocyte development, for which its structure and its composition are specialized, providing the necessary microenvironments for each step of T cell differentiation and selection (1, 2). In a mature thymus, developing thymocytes compose around 99% of the thymus cellularity (3), meaning that the thymus stroma, which comprises all the non-hematopoietic cellular components of the thymus, accounts for less than 1% of the cells found in the thymus (4, 5). The majority of stromal cells consist of thymic epithelial cells (TECs), which not only provide the three-dimensional matrix in which T cells develop but also control the homing, expansion, maturation, and selection of these thymocytes (4, 6, 7).

The mature thymus can be anatomically subdivided in two main regions: the peripheral cortex and the inner medulla (1, 6, 7), that are conserved throughout evolution (4). This allows the classification of the TECs in cortical (cTECs) and medullary (mTECs), which have differential morphological, functional, and antigenic properties (4, 6). Both mTECs and cTECs derive from a common bipotent TEC progenitor that expresses MHC class I, MHC Class II, EpCAM, and intracellular keratins (4). However, they express distinct cortical (cytokeratin-8^+^ and -18^+^, Ly51^+^), and medullary (cytokeratin-5^+^ and -14^+^, Ly51^−^) markers that, together with the mTEC-specific ability to bind the *Ulex europaeus* lectin agglutinin (UEA-1), allow them to be distinguished (1, 4, 8). mTECs can be further subdivided in different subpopulations by the expression of MHCII and the accessory molecules, such as CD40 and CD80/86, with AIRE expression being found specifically in a subpopulation of MHCII^high^, CD80/86^high^ mTECs (9, 10). All these subsets of TECs are highly specialized to provide the cytokines, chemokines, lineage inductive ligands, selective self-antigens, cell surface molecules, and extracellular matrix elements necessary for T cell development, which makes this process strictly dependent on the communication between TECs and the developing T cells (11, 12).

Allogeneic bone marrow transplantation (BMT) is currently the most effective treatment for lymphoid and myeloid cancers as well as to treat genetic immune disorders and various autoimmune disorders (13). Prior to transplantation, a patient must undergo a combination of conditioning or preparative regimes, normally consisting of radiotherapy (frequently in combination with chemotherapeutic drugs), in order to eliminate endogenous HSC and resident host immune cells (14–16). Ionizing radiation (IR) causes many deleterious and dose-dependent effects on the hematopoietic system, which is highly radio-sensitive and is one of the first systems to collapse following exposure to IR (17, 18). However, other cell types such as TECs are also vulnerable to damage inflicted during the BMT process by agents, such as radiation or chemotherapy (19). In order for a BMT to be successful, not only the presence of viable progenitors is necessary but also the maintenance of a functional microenvironment to support differentiation of these cells is crucial (20). This deleterious effect on the thymus functionality is one of the main causes that has been hypothesized to explain the prolonged periods of T-cell deficiency that BMT patients often suffer and that render them highly susceptible to common and opportunistic infections, as well as occurrence and relapse of cancers (19, 21). For this reason, investigation of the effects that ionizing radiation causes on TECs and their ability to perform their normal function is crucial for improving the outcomes of BMT.

Ionizing radiation causes extensive damage to the genome of the cells, either by direct energy transfer to the DNA or most frequently trough the generation of free radicals by ionization of molecules, primarily water. Of all lesions induced, DNA double strand breaks (DSBs) are the most genotoxic due to their difficulty to be repaired (18, 22). This destructive impact on genomic integrity triggers the activation of the DNA damage response (DDR), which is a complex signaling network that allows the cells to mount an orchestrated response to damage in their DNA (23). The DDR is composed of sensors that monitor DNA for structural abnormalities (damaged DNA), transducers that transmit and amplify the damage signal, and effectors in charge of triggering and coordinating biological processes. Such processes include transient cell cycle arrest (checkpoints), DNA repair, alteration of transcriptional programs, apoptosis, or senescence (24, 25).

We have previously shown how the execution of the DDR can have a profound impact on the cells sensitivity to IR (26). Here, we characterized the DDR of TEC lines in order to identify the main mechanisms underlying their survival after IR and compared the specific responses of cortical and medullary TECs. Since we previously demonstrated a role of hypoxia in enhancing the DDR of mesenchymal stromal cells (27), we also analyzed whether hypoxia plays a role in regulating TEC response to IR. We show how exposure to IR has a profound effect on primary mouse TEC functionality by markedly decreasing their expression of factors that are essential for their functions. To the best of our knowledge, this is the first time that the DDR of TECs has been studied in detail.

## Materials and Methods

### Cell Culture and Treatment

The cortical thymic epithelial cell line cTEC 1–2 and the medullary thymic epithelial cell line mTEC 3–10 were kindly provided by Prof. Georg Holländer (Department of Biomedicine, University of Basel) and ST4.5 CD4^+^ CD8^+^ thymocyte cell line was provided by Dr. Anne Wilson (Ludwig Institute of Cancer Research, Lausanne). All cell lines were cultured in Dulbecco’s modified Eagle’s medium high glucose (Gibco) supplemented with 10% fetal bovine serum (Sigma-Aldrich) and 1% penicillin/streptomycin sulfate solution (Gibco).

All cell types were continuously cultured in humidified incubators at 37°C containing 21% O_2_ (normoxia) or 5% O_2_ (hypoxia) for at least 1 week prior to experimentation.

Cells were γ-irradiated at the indicated doses using a Maintenance Millennium Sample Irradiator containing a ^137^Cs source at a dose rate of approximately 102 cGy/min. Cells were treated with 1µM staurosporine solution (Cell Signaling Technologies) or 25 µM 2-bromodeoxiuridine (BrdU) and harvested at the indicated time points post-treatment.

### Mice

C57BL/6 mice were bred under pathogen-free conditions at the Center for Biomedicine at the University of Basel. All animal experiments were carried out within institutional guidelines (authorization numbers 1886 and 1888 from Kantonales Veterinäramt, Basel).

### Isolation and Sorting of Mouse TEC Subpopulations

Two groups of 20 C57BL/6 mice were used in this experiment. One of the groups was irradiated with 9 Gy, while the other was left untreated as control. Twenty-four hours after irradiation, thymic stromal cells were isolated from the 20 control and 20 irradiated thymi and sorted according to their cell surface phenotypes following the protocol described in the Methods in Supplementary Material. Sorted cells were pelleted, resuspended in 500 µl TRIzol (Life Technologies), and stored at −20°C for further processing.

### Growth Curve Analysis

Cells were seeded into six-well plates (Nunc) at a concentration of 50,000 cells/well. Individual cultures were harvested daily for 7 days, and cell counts were performed in duplicate in a hemocytometer using trypan blue exclusion of dead cells.

### Clonogenic Survival Assay

Adherent TEC cell lines were irradiated at 1–8 Gy and seeded into six-well plates (Nunc) at a concentration of 200 cells per well. Cells were incubated for 8 days until colonies were clearly visible. Colonies were stained with Coomassie Blue (Sigma-Aldrich) and counted. All colony images are representative of one of four independent experiments. Non-adherent ST4.5 cells were irradiated with 1–8 Gy, seeded into six-well plates (Nunc) at a concentration of 30,000 cells per well, and harvested 5 days postirradiation. Cell numbers were counted in duplicate using a hemocytometer, and trypan blue exclusion of dead cells was performed. The percentage survival of each cell type was determined by normalizing the number of colonies/cells generated by irradiated cultures to the number of colonies/cells generated by control un-irradiated cultures.

### Flow Cytometry

Cells were trypsinized to obtain a single-cell suspension, filtered trough a 30-µm filter (Cell Trics), and counted prior to staining following the different protocols described in the Methods in Supplementary Material. Cells were then analyzed using BD FACS Canto^®^ or BD Accuri™ C6 flow cytometers (BD Biosciences) and FlowJo^®^ software (TreeStar Inc., OR, USA). Details of all antibodies used can be found in the Methods in Supplementary Material.

### Western Blotting

Whole-cell extracts were prepared from control or irradiated cells at the indicated time points postirradiation by direct addition of 1× Laemmli buffer to the cells still adhered to the culture plates, following one wash with ice-cold PBS. Cells were disaggregated into the Laemmli buffer using a cell scraper, heated at 95°C for 5 min, and sonicated prior to separation using SDS-PAGE gels and transferred to nitrocellulose membranes. Chemiluminescence was detected using SuperSignal West Pico Chemiluminescent Substrate (Thermo Scientific) and medical X-ray film (Konica Minolta Medical & Graphic Imaging Inc.). In assays in which protein quantification was necessary, this was performed using a LiCor Odissey infrared imaging system according to manufacturer’s instructions. Details of all antibodies used can be found in the Methods in Supplementary Material.

### Immunofluorescence Microscopy

Cells were cultured on glass coverslips in 21 or 5% O_2_ for 48 h prior to irradiation. All cultures were fixed in 4% paraformaldehyde (Sigma-Aldrich), permeabilized in 0.1% Triton^®^-X 100 solution and nuclei stained for γH2AX and Rad51 IR-induced foci (IRIF) as previously described [Sugrue et al. (24, 26)]. All images were captured using 40× or 60× magnification on a Delta Vision integrated microscope system (Applied Precision) controlled by SoftWoRx software mounted on an IX71 Olympus microscope. Images were deconvolved using the ratio method and maximal intensity projections obtained using SoftWoRx. All images shown are representative of one of five independent experiments. The number of γH2AX and Rad51 IRIF per nucleus was quantified blind using customized macros for ImageJ in a total of 50 cells per time point in each experiment. Details of all antibodies used can be found in the Methods in Supplementary Material.

### qPCR

Total RNA was isolated from cells by TRIzol^®^ Reagent (Life Technologies)–chloroform extraction. cDNA was generated using Applied Biosystems’ High-Capacity cDNA Reverse Transcription Kit according to the manufacturer’s instructions. The resulting cDNA was used as a template in quantitative PCR reactions with specific primers on a Step One Plus Real-Time PCR System (Applied Biosystems). The reactions were prepared with SYBR Select reaction mix from Applied Biosystems. Predesigned KiCqStart^®^ primer pairs for mouse Aire, Dll4, Flt3l, Il-7, Kitl, β5t, Ctss, Ccl17, Ccl19, Ccl21, Ccl22, Ccl25, Xcl-1, Cxcl12, Bim, β-Actin, and Gapdh were purchased from Sigma-Aldrich. Gene expression analysis was carried out using the 2^−ΔΔCt^ method and β-Actin and Gapdh were used as control genes for normalization.

### Whole Thymic Stroma Gene Expression Analysis

C57BL/6 mice were irradiated with 9 Gy, and thymic stroma was subsequently obtained from control and irradiated thymi. T cells were depleted by gently pressing thymuses through a 70-µm pore size cell strainer followed by several washes with ice-cold PBS. The remaining stroma was then fragmented and disaggregated in TRIzol^®^ Reagent (Life Technologies) for RNA isolation using the TRIzol–chloroform method. Resulting RNA was used as template for cDNA synthesis using the High-Capacity cDNA Reverse Transcription Kit from Applied Biosystems according to the manufacturer’s instructions, and qPCR reactions were performed as described above. Gene expression analysis was carried out separately for each technical replicate using the 2^−ΔΔCt^ method, prior to averaging, as described in Ref. (28), and SEM of both control and irradiated samples are reported. Gapdh was used as endogenous control gene, and untreated samples were used as reference for normalization.

### Primary Mouse TEC Subpopulations Gene Expression Analysis

RNA was isolated from irradiated or control-sorted TEC subpopulations using the TRIzol–chloroform method. cDNA was then synthetized using either the High-Capacity cDNA Reverse Transcription Kit from Applied Biosystems or the QuantiTect Whole Transcriptome Amplification Kit from Quiagen, according to the manufacturer’s instructions. qPCR reactions were performed as described above.

### DDR qPCR Arrays

RNA was isolated from TEC cell lines cultured in normoxia (21% O_2_) or hypoxia (5% O_2_) using the TRIzol–chloroform method. Five hundred nanograms per sample of the resulting total RNA were used as a template for cDNA synthesis using Quiagen’s RT2 First Strand Kit according to the manufacturer’s protocol. qPCR reactions were prepared using the RT2 SYBR Green ROX qPCR Mastermix from Quiagen and loaded into the commercial customized Mouse DDR RT2 Profiler PCR Arrays which include primers for DNA Ligase IV, Bcl-2, Bcl-XL, and Puma in addition to the 84 DDR genes present in the standard PCR arrays.

## Results

### Characterization of TEC Lines

In order to study the responses of TEC lines to ionizing irradiation, we used the cell lines TEC 3–10 (medullary TEC) and TEC 1–2 (cortical TEC). These cell lines were originally established by Mizuochi et al. from C56BL/6 mice, who characterized their medullary and cortical nature by immunostaining with the Th-3 and Th-4 antibodies (29). Prior to our experiments, we verified the phenotype of these cells by morphological (Figure S1A in Supplementary Material) and flow cytometric analysis of CD45, EpCAM, Ly51, and MHC-II surface marker expression and binding of the UEA-1 lectin. Thus, we were able to confirm the identity of the mTEC 3–10 cell line as CD45^−^ EpCAM^+^ Ly51^−^ UEA-1^+^ MHC-II^+^ and the cTEC 1–2 cell line as CD45^−^ EpCAM^+^ Ly51^+^ UEA-1^−^ MHC-II^+^ (Figures S1B,C in Supplementary Material).

### TEC Lines Are Resistant to Ionizing Radiation, and Hypoxia Reduces mTEC Radio-Resistance *In Vitro*

**Figure 1:**
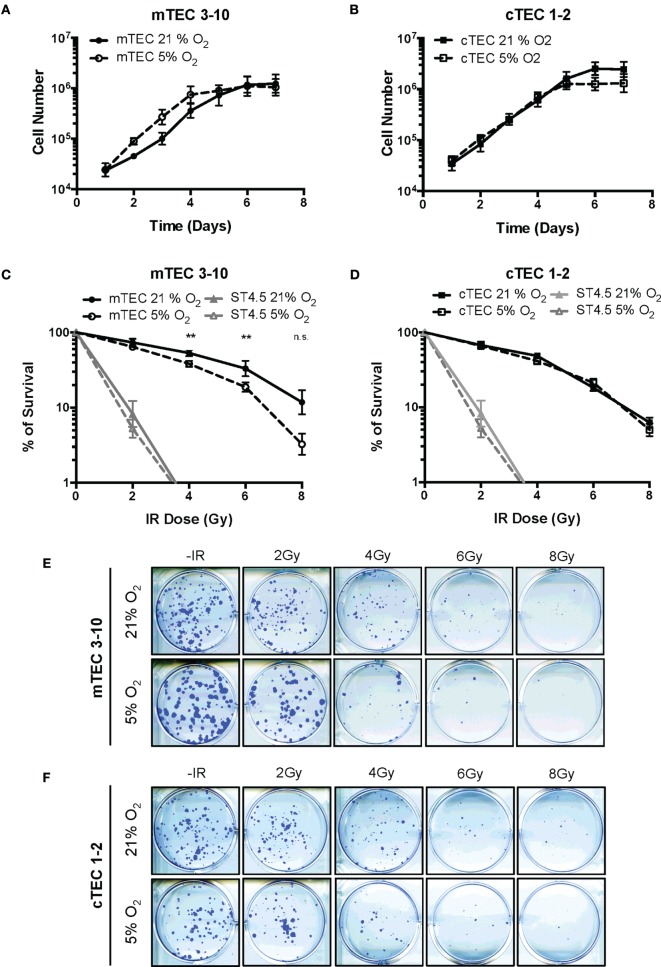
**Thymic epithelial cell (TEC) survival to ionizing radiation (IR)**. Growth curves of **(A)** mTEC 3–10 and **(B)** cTEC 1–2 cell lines cultured in 21/5% O_2_. Clonogenic survival assays of **(C)** mTEC 3–10 and ST4.5 cells and **(D)** cTEC 1–2 and ST4.5 cell lines in 21 or 5% O_2_. **p < 0.01 compared with normoxic samples, two-way ANOVA. Representative images of **(E)** mTEC and **(F)** cTEC colonies generated in clonogenic survival assays following treatment with 0, 2, 4, 6, and 8 Gy.

To determine how hypoxia influenced cell growth, TEC lines were cultured in either normoxia (21% O_2_) or hypoxia (5% O_2_) and growth curves plotted. A tendency for enhanced growth of mTEC 3–10 cells was observed under hypoxic conditions (with an average doubling time of 18.29 h in normoxia and 14.55 h in hypoxia), (Figure 1A) whereas cTEC 1–2 cells grew at a similar rate in both hypoxia and normoxia (17.41 h doubling normoxia and 17.32 h in hypoxia) (Figure 1B). Interestingly, cTEC 1–2 cells grew to higher cell number (~twofold) under normoxic conditions, whereas mTEC 3–10 cells, despite faster growing in hypoxia, reached the same plateau cell concentration in both conditions. To study the effects of irradiation on cell lines, clonogenic survival assays were carried out. Results of actual colonies obtained are shown for mTEC and cTEC in Figures 1E,F, respectively. Confirming their relative enhanced growth in hypoxia, colony sizes of mTEC 3–10 cells were detectably larger in hypoxia, although there were fewer colonies. For cTEC 1–2 cells, there was no observable change in colony size in hypoxia and colony numbers seemed unchanged. Results of a series of such experiments are shown for mTEC 3–10 and cTEC 1–2 cells in Figures 1C,D, respectively, and a comparison of both TEC cell lines can be found in Figure S2 in Supplementary Material. In these experiments, the ST4.5 CD4/CD8 double positive (DP) T cell line was included as a radio-sensitive control [Sugrue et al. (24, 26)]. Both mTECs and cTECs showed a much higher radio-resistance than the DP cell line ST4.5. Both mTEC 3–10 and cTEC 1–2 lines showed a very similar survival to low IR doses; however, mTEC 3–10 cells show an increased radio-resistance to the highest doses of IR (particularly noticeable at 6–8 Gy) (Figure S2 in Supplementary Material). Taken together, these results indicate that mTEC 3–10 cells are more resistant to high doses of IR and that hypoxia specifically reduces the radio-resistance of this cell line. Our clonogenic survival assays also showed that both cTEC 1–2 and mTEC 3–10 cell lines retained approximately 50% of their colony formation capacity after treatment with 3 Gy compared to the untreated condition (Figures 1C,D); therefore, this dose was chosen for most of the subsequent experiments.

### Oxygen Level Does Not Affect the Cell Cycle Regulation of TECs

**Figure 2:**
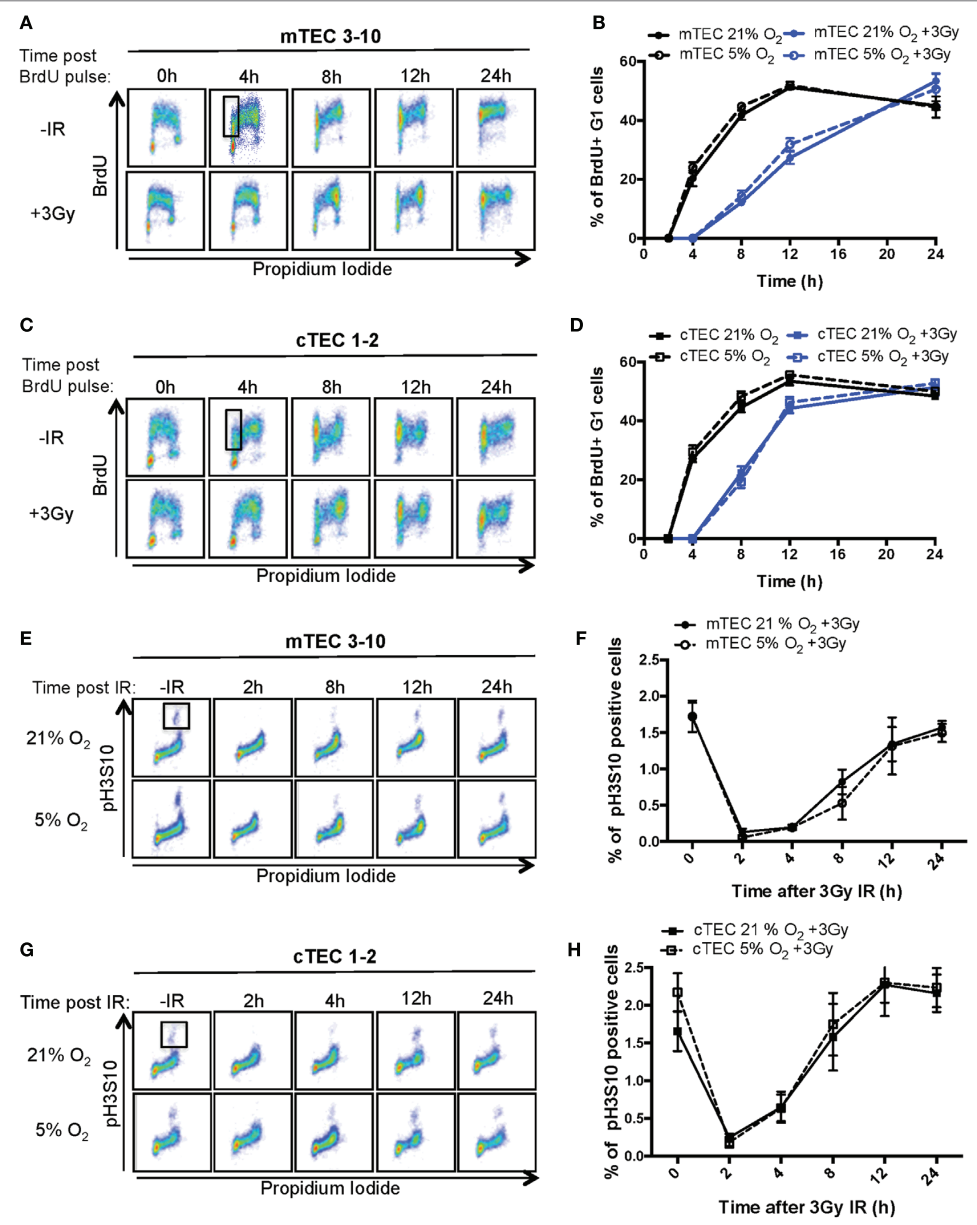
**Thymic epithelial cell (TEC) cell cycle regulation and checkpoint analysis**. Cytograms of **(A)** mTEC 3–10 and **(C)** cTEC 1–2 cells stained for bromodeoxiuridine (BrdU) incorporation and DNA content (propidium iodide) cultured in 21% at different time points following BrdU pulse, with or without treatment with 3 Gy of ionizing radiation (IR). Representative gating strategy for the identification of BrdU^+^ G1 cells is shown in black. Quantification of average percentage of BrdU-labeled G1 phase cells in **(B)** mTEC 3–10 and **(D)** cTEC 1–2 cells cultured in either 21 or 5% O_2_, 0–24 h post BrdU pulse, with or without treatment with 3 Gy of ionizing radiation (IR). Cytograms of **(E)** mTEC 3–10 and **(G)** cTEC 1–2 cells stained for histone H3 Ser10 phosphorylation (pH3S10) and DNA content (propidium iodide) in 21 or 5% O_2_ at different time points following treatment with 3 Gy of IR. Representative gating strategy for the identification of pH2S10^+^ cells is shown in black. Quantification of average mitotic index (% of pH3S10 positive cells) in **(F)** mTEC 3–10 and **(H)** cTEC 1–2 cells, 0–24 h postirradiation.

In response to genotoxic lesions such as those introduced by IR, cells activate the DDR, a complex signaling network that orchestrates the cellular response to such lesions. One of the cell’s earliest responses to DNA damage is to induce a cell cycle arrest (30). To study the cell cycle checkpoints activated by TECs in response to IR, cell cycle progression of BrdU pulse labeled mTECs and cTECs was analyzed by flow cytometry. Thus, combined BrdU and PI staining allows to distinguish cells in G1, S, and G2/M phases of the cell cycle as well as progression of BrdU-labeled (S phase) cells through the cell cycle and their return to the G1 phase (Figures 2A–D; Figure S2B in Supplementary Material). After receiving a 3Gy IR treatment, both mTEC 3–10 and cTEC 1–2 cells accumulated in G2/M phase until about 8 h, which indicates a strong prevalence of the G2/M checkpoint in these cells, with very little or no activation of the G1 or intra-S checkpoints (Figures 2A,C). As the cell cycle progresses, a subpopulation of newly formed BrdU-labeled G_1_ cells appears and increases in size. Quantification of this new subpopulation was used as readout for the kinetics with which cells resumed the cell cycle after the genomic insult and left the G2/M arrest. The delay in cell cycle progression induced by IR can be clearly observed in comparison with the untreated cells (Figures 2A–D), although no differences were detected between normoxic and hypoxic conditions for both mTEC 3–10 and cTEC 1–2 cell lines (Figures 2B,C; Figure S2B in Supplementary Material). However, comparison between mTEC 3–10 and cTEC 1–2 cells evidenced a faster recovery from the cell cycle arrest in cTEC 1–2 cells than in mTEC 3–10, as evidenced by the higher proportion of BrdU-positive G1 cells present 8 and 12 h after IR (Figures 2B,D).

Since both TEC cell lines seem to mainly rely on the G_2_/M checkpoint, and because of the fact that the BrdU/PI assay does not allow the discrimination between G_2_ and M phases of the cell cycle, a G2/M checkpoint assay was used. To do so, a mitotic index analysis was performed flow cytometrically using combined intracellular staining for phosphorylated histone H3 Serine10 (pH3S10) and PI. The pH3S10 phosphorylation is a mark of chromosomal condensation and is broadly used to identify mitotic cells. In response to IR, the activation of the G2/M checkpoint results in the arrest of cells in G2 and the consequent loss of the mitotic cell population (Figures 2E,G; 2 h time point). Only after several hours (4 h for cTEC 1–2 and 8 h for mTEC 3–10), cells begin to resume the cell cycle and mitotic cells begin to be detectable again. This difference between the timing with which mTEC 3–10 and cTEC 1–2 cells resume mitosis indicates a distinct cell cycle regulation between the two cell types, with cTEC 1–2 cells releasing from the G2/M arrest faster than mTEC 3–10 cells. However, the quantification of the mitotic index did not show any significant difference between normoxia and hypoxia (neither for cTEC 1–2 nor for mTEC 3–10 cells) (Figures 2F,H), indicating that oxygen levels do not affect cell cycle regulation in these cells.

### Hypoxia Does Not Influence the DSB Repair Capacity of TEC Lines

**Figure 3:**
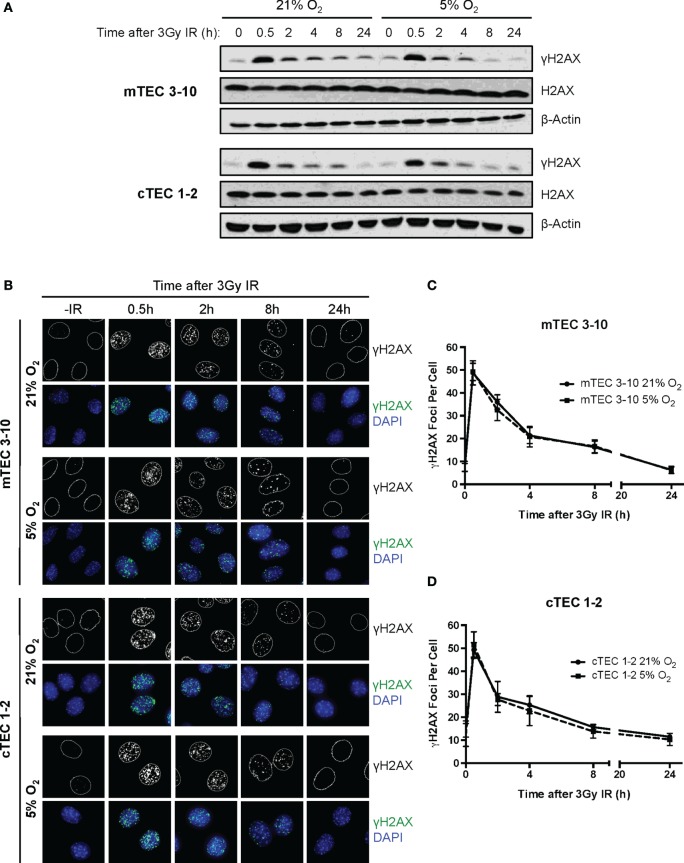
**Double strand break repair kinetics in thymic epithelial cells (TECs)**. **(A)** Representative western blots showing γH2AX and β-Actin levels in mTEC 3–10 and cTEC 1–2 cells cultured in 21 or 5% O_2_, 0–24 h after irradiation with 3 Gy. **(B)** Representative images of mTEC 3–10 and cTEC 1–2 nuclei stained for γH2AX IR-induced foci (IRIF), in 21 or 5% O_2_, 0–24 h post 3Gy irradiation. Average number of γH2AX IRIF per nucleus in **(C)** mTEC 3–10 cells and **(D)** cTEC 1–2 cells, 0–24 h post-ionizing radiation (IR), *n* = 5.

**Figure 4:**
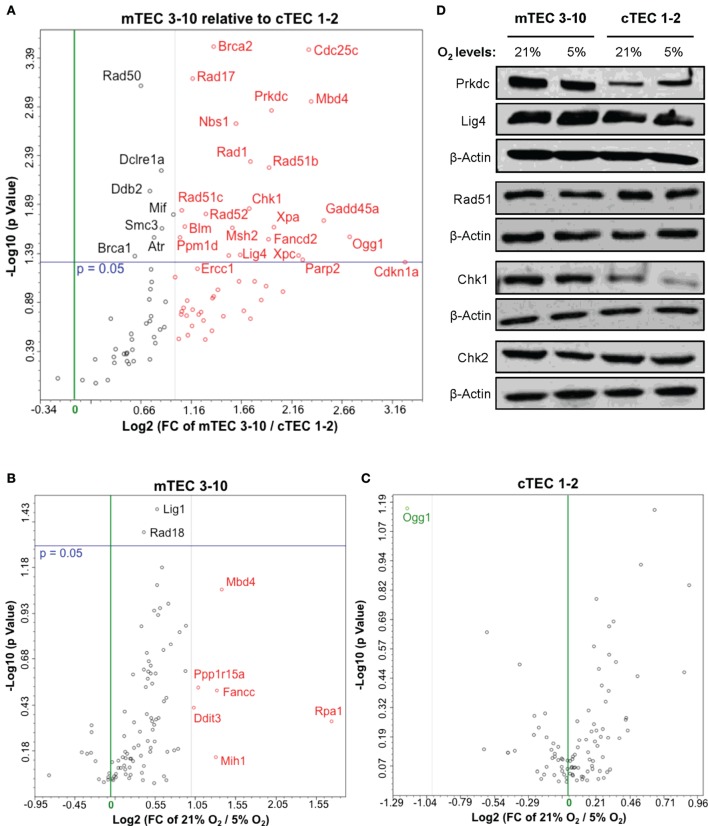
**Thymic epithelial cell (TEC) DNA damage response gene expression analysis**. Volcano plots of qPCR array data comparing **(A)** normoxic mTEC 3–10 vs. cTEC 1–2; **(B)** normoxic vs. hypoxic mTEC 3–10; and **(C)** normoxic vs. hypoxic cTEC 1–2 gene expression. Green and black vertical lines represent 0 and twofold expression changes, respectively. Blue horizontal lines represent a *p* value of 0.05, with significantly regulated genes being shown above them. All genes upregulated more than twofold are shown in red, while all genes downregulated more than twofold are shown in green (independently of their statistical significance). **(D)** Representative western blots showing mTEC 3–10 and cTEC 1–2 expression levels of DNA-PKcs, DNA Ligase IV, Rad51, Chk1, Chk2, and β-Actin in normoxia (21% O_2_) and hypoxia (5% O_2_).

In light of the decreased radio-resistance of mTEC 3–10 cells in hypoxia, we wondered whether the DNA repair capacity might be altered in this condition. The phosphorylation of Ser139 of the histone variant H2AX (γH2AX) was used as a marker of unrepaired DSBs by both western blotting and immunofluorescence analysis (Figures 3A–C) in order to determine the kinetics of DSB repair. In both mTEC 3–10 and cTEC 1–2, the highest levels of γH2AX phosphorylation were observed 30 min after IR, with a progressive decrease consistent with DSB repair. The quantification of the number of IR-Induced γH2AX foci IRIF showed no significant difference between normoxic and hypoxic mTEC 3–10 or cTEC 1–2 cells (Figures 3C,D), indicating that hypoxia does not have significant effects in the DSB repair capacity of the cells. Consistently with this observation, quantification of Rad51 IRIF, a direct mark of DNA DSB repair by homologous recombination, also did not show any significant difference between normoxia and hypoxia (Figure S3 in Supplementary Material). In line with this observation, western blot analysis of the levels of expression of different DDR factors (DNA-PKcs, DNA Ligase IV, Rad51, Chk1, and Chk2) showed only cell type-related differences (higher expression of the NHEJ factors DNA-PKcs and DNA Ligase IV and the effector kinase Chk1 by mTEC 3–10 than cTEC 1–2) but no effect caused by the hypoxic treatment on the cells (Figure 4D).

### mTECs Express Higher Levels of DDR Factors and Exposure to Hypoxia Results in Their Downregulation

Our results so far evidence some differences in the DDR of medullary and cortical TEC lines, as well as normoxic and hypoxic mTEC 3–10 cells. To further characterize the DDR components of each cell type, a comprehensive analysis of the gene expression of an array of 87 genes belonging to the DDR signaling network was performed using commercial qPCR arrays. Comparison of the gene expression of mTEC 3–10 and cTEC 1–2 cells showed a marked trend toward higher levels of expression of DDR genes in mTEC 3–10 cells compared to cTEC 1–2. Although approximately 60% of the genes showed greater than twofold increase in mRNA expression in mTEC 3–10 cells (Figure 4A, shown in red; Table 1), only approximately 35% of all the genes analyzed were significantly more expressed (*p* value > 0.05) in mTEC 3–10 cells (Figure 4A; Table 1). This finding may indicate the presence of a more robust DDR in mTEC 3–10 cells than in their cortical counterparts. Among the significant differentially regulated genes, mTEC 3–10 cells showed enrichment in DNA DSB repair factors involved in HR (Rad51b, Rad51c, Rad52, Fancd2, Blm, Brca1, and Brca2) and NHEJ, such as Prkdc (DNA-PKcs) and Lig4 (confirming the western blot results), as well as key players involved in excision repair pathways such as Parp2, Ddb2, Xpa, Xpc, Ercc1, and Gadd45a. mTEC 3–10 cells also showed higher levels of genes involved in sensing and coordinating the DDR, such as Nbs1, Rad50, Chk1, and Atr, and also cell cycle regulation such as Cdkn1a (p21) and Cdc25c, which may explain the differential checkpoint regulation observed between the two cell lines (Figure 2). Western blot analyses of DDR factors showed that this regulation is also maintained at the level of protein for at least some of the transcripts analyzed (Figure 4D). Consistently with the previous qPCR data, mTEC 3–10 cells express higher protein levels of DNA-PKcs, DNA Ligase IV, and Chk1 than cTEC 1–2 cells, but no difference was observed for the HR factor Rad51 or the other main effector kinase Chk2.

**Table 1:** **Thymic epithelial cells (TEC) DNA damage response gene expression analysis**.

mTEC 21% O_2_ relative to cTEC 21% O_2_			mTEC 21% O_2_ relative to mTEC 5% O_2_			cTEC 21% O_2_ relative to cTEC 5% O_2_		
Gene symbol	Fold regulation	*p*-Value	Gene symbol	Fold regulation	*p*-Value	Gene symbol	Fold regulation	*p*-Value
Cdkn1a	**9.7867**	**0.049561**	Rpa1	**6.6947**	0.4525	Ogg1	−2.2717	0.068328
Ogg1	**6.69**	**0.027358**	Mbd4	**2.6039**	0.086309			
Gadd45a	**5.5777**	**0.018754**	Fancc	**2.4879**	0.306775			
Mbd4	**5.112**	**0.001134**	Mlh1	**2.4701**	0.709673			
Cdc25c	**5.0346**	**0.000334**	Ppp1r15a	**2.1235**	0.296088			
Parp2	**4.83**	**0.047262**	Ddit3	**2.0463**	0.380959			
Xpc	**4.698**	**0.042352**	Lig1	1.4897	**0.031506**			
Ppp1r15a	**4.2181**	0.09951	Rad18	1.3287	**0.042163**			
Xpa	**3.9535**	**0.021999**						
Prkdc	**3.8944**	**0.001389**						
Rad51b	**3.8334**	**0.005391**						
Fancd2	**3.8197**	**0.029257**						
Fancg	**3.7531**	0.0874						
Abl1	**3.6328**	0.121405						
Xrcc1	**3.4676**	0.078948						
Rad1	**3.3607**	**0.004642**						
Chek1	**3.3356**	**0.014122**						
Fen1	**3.2154**	0.157843						
Lig4	**3.1376**	**0.042301**						
Trp53	**3.1171**	0.078992						
Nbs1	**3.043**	**0.001907**						
Msh2	**2.9736**	**0.022276**						
Ercc1	**2.9049**	**0.042759**						
Bcl2	**2.7883**	0.177894						
Xrcc2	**2.6866**	0.096181						
Brca2	**2.618**	**0.00031**						
Apex1	**2.6124**	0.11235						
Ung	**2.5834**	0.252669						
Rad52	**2.4769**	**0.016033**						
Rpa1	**2.4658**	0.305147						
Rnf8	**2.4384**	0.197479						
Trp53bp1	**2.3941**	0.161481						
Rad9a	**2.3515**	0.170914						
Rad21	**2.3323**	0.058508						
Xrcc3	**2.2615**	0.152825						
Rad17	**2.2565**	**0.000658**						
Topbp1	**2.2545**	0.223298						
Atm	**2.1819**	0.277075						
Hus1	**2.1729**	0.122838						
Nthl1	**2.1645**	0.174959						
Blm	**2.1445**	**0.02165**						
Msh3	**2.1212**	0.147084						
Rad51c	**2.0965**	**0.014677**						
Mpg	**2.0873**	0.170764						
Ppm1d	**2.0701**	**0.027739**						
Pole	**2.0584**	0.300385						
Mdc1	**2.0072**	0.071037						
Mif	1.9804	**0.016166**						
Smc3	1.8309	**0.022549**						
Dclre1a	1.825	**0.005796**						
Atr	1.7345	**0.027855**						
Ddb2	1.6814	**0.009335**						
Rad50	1.5847	**0.000778**						
Brca1	1.5164	**0.043145**						

*List of genes showing greater than twofold up- or downregulation and/or *p* value lower than 0.05 in normoxic mTEC 3–10 vs. cTEC 1–2 (first column), normoxic vs. hypoxic mTEC 3–10 (second column) and normoxic vs. hypoxic cTEC 1–2 cells (third column). *p* values lower than 0.05 and expression fold changes greater than 2 are highlighted in bold*.

When comparing the effects of hypoxia on each cell line, mTEC 3–10 cells seem to be more responsive to the hypoxia treatment, showing a marked trend toward a downregulation of most of the genes when exposed to low oxygen levels (Figure 4B). However, only six genes show a greater than twofold upregulation in normoxia compared to hypoxia (Figure 4B, shown in red; Table 1) and only two genes (Lig1 and Rad18) showed a modest but significant upregulation (Figure 4B; Table 1). In contrast, culturing cTEC 1–2 cells in hypoxia did not induce many changes in expression of genes involved in the DDR pathway, with only one gene upregulated (Ogg1) over twofold but showing no statistical significance (Figure 4C, shown in green; Table 1).

### Hypoxia Promotes mTEC Apoptosis upon Irradiation through the Upregulation of Bim

**Figure 5:**
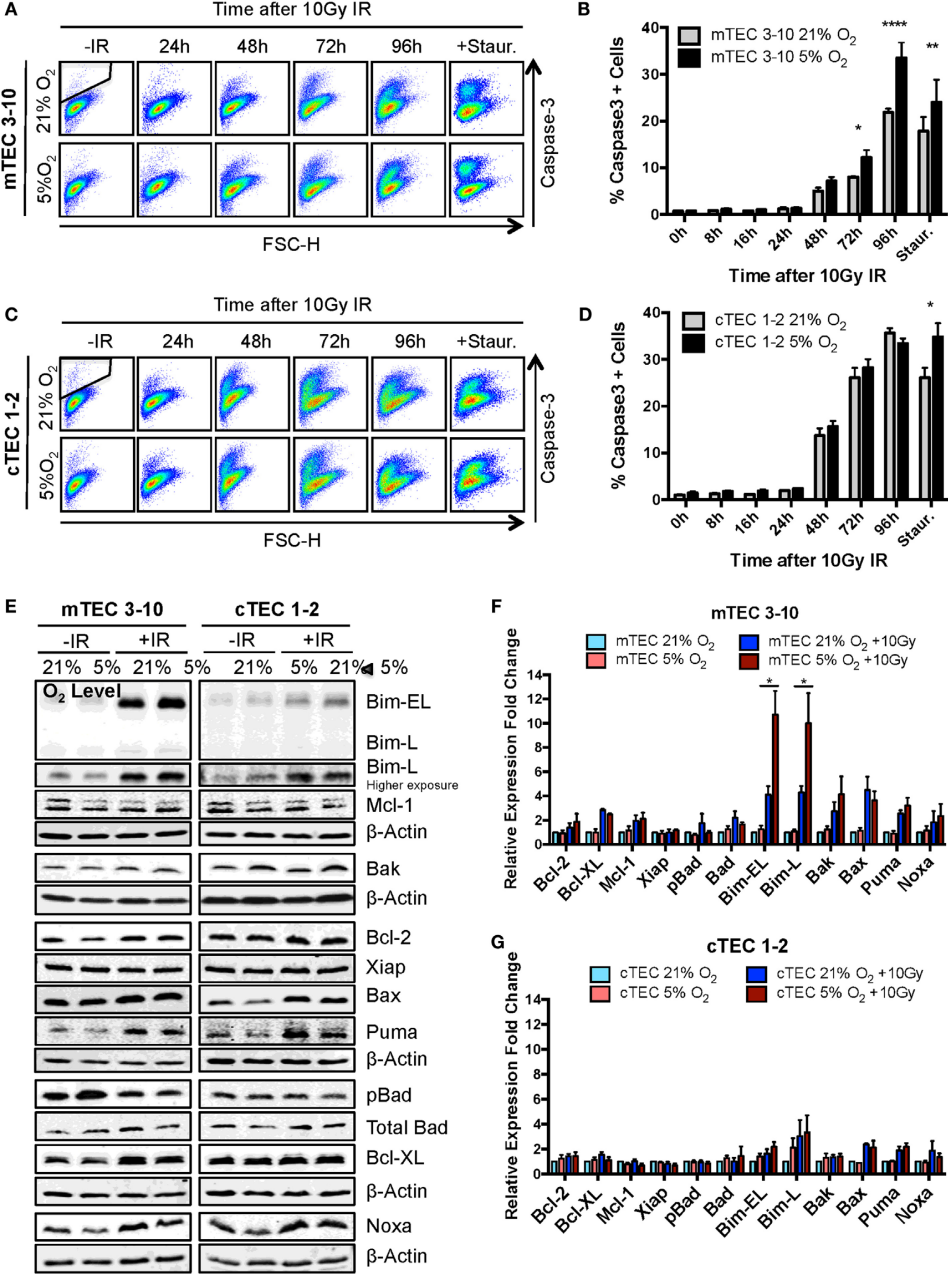
**Ionizing radiation (IR)-induced apoptosis analysis in thymic epithelial cells (TECs)**. **(A)** Representative cytograms of mTEC 3–10 cells stained for cleaved Caspase-3, and **(B)** quantification of average percentage of Caspase-3 positive mTEC 3–10 cells, 0–96 h post 10 Gy of IR. Staurosporin treatment was used as a positive control for the activation of the apoptosis pathway. Representative gating strategy for the identification of Caspase-3^+^ cells is shown in black. *p < 0.05, **p < 0.01, ****p < 0.0001 compared to normoxic samples, two-way ANOVA, *n* = 4. **(C)** Representative cytograms of cTEC 1–2 cells stained for cleaved Caspase-3, and **(D)** quantification of average percentage of Caspase-3 positive cTEC 1–2 cells 0–96 h following treatment with 10 Gy of IR. *p < 0.05, **p < 0.01, ****p < 0.0001 compared to normoxic samples, two-way ANOVA, *n* = 4. **(E)** Representative western blots and quantification of **(F)** mTEC 3–10 and **(G)** cTEC 1–2 expression level of pro- and anti-apoptotic proteins. β-Actin was used as reference gene for the quantification and all values were normalized against the untreated normoxic samples. *p < 0.05, multiple *t*-tests with Holm–Sidak posttest correction, *n* = 4.

Since the decreased radio-resistance of mTEC 3–10 cells in hypoxia does not seem to be related to differences in repair capacity of DNA lesions or differential regulation of cell cycle checkpoints, we wondered whether this could be due to an enhanced susceptibility to undergo apoptosis in response to IR. Apoptosis was measured by cleaved Caspase-3 staining and flow cytometric analysis at different times up to 96 h following irradiation with 10 Gy and using staurosporine treatment as positive control. In contrast with previous experiments, a higher IR dose of 10 Gy was chosen in this case in order to efficiently study cell death rather than repair of the DNA lesions. cTEC 1–2 cells showed higher sensitivity to IR as evidenced by the faster increase in Caspase-3 positive cells, reaching 30% after 72 h, when only 10% of mTEC 3–10 cells had activated the apoptotic pathway (Figures 5A–D). Hypoxic mTEC 3–10 cells showed a faster accumulation of Caspase-3 positive apoptotic cells over time, with significant differences being observed at 72 and 96 h after IR (Figures 5A,B). Consistent with this, significantly higher apoptotic rates were also observed in hypoxic mTEC 3–10 upon treatment with staurosporine. In contrast, cTEC 1–2 cells only showed higher apoptosis in hypoxia when treated with staurosporine, but not following IR at any of the time points analyzed (Figures 5C,D). This result correlates with those from the clonogenic survival assays previously described.

In order to investigate the mechanism underlying the increased propensity of hypoxic mTEC 3–10 cells to undergo apoptosis, the level of different pro- and anti-apoptotic proteins was analyzed by western blotting. mTEC 3–10 and cTEC 1–2 cells showed differential responses to IR in terms of their regulation of apoptotic factors. Whereas in mTECs, there is a higher induction in expression of the pro-apoptotic proteins Bim, Bax, Bak, Noxa, and Puma upon irradiation, this is also accompanied by an increase in the levels of anti-apoptotic proteins, such as Bcl-XL or Bcl-2 (Figures 5E,F). This induction of anti-apoptotic proteins may counteract the effect of the increase in pro-apoptotic factors. In contrast, cTEC 1–2 cells show a less pronounced IR-induced increase in the levels of pro-apoptotic factors, and a very mild induction of anti-apoptotic proteins (Figures 5E,G). This differential response may explain the previously observed higher sensitivity of cTEC 1–2 cells to IR-induced apoptosis.

Interestingly, while most of the apoptotic factors studied followed the same pattern of expression in normoxia and hypoxia for both TEC cell lines, hypoxic mTEC 3–10 cells showed a significantly higher induction of the expression of Bim after IR (both Bim-EL and Bim-L isoforms) (Figures 5E,F). This specific increase in hypoxia is not accompanied by an increase in the levels of any anti-apoptotic protein that could counteract the effect of Bim, and this may be the key to the greater propensity of mTEC 3–10 cells to undergo apoptosis under hypoxic conditions.

### Ionizing Radiation Profoundly Affects Expression of Functional Factors in Primary Mouse TECs

**Figure 6:**
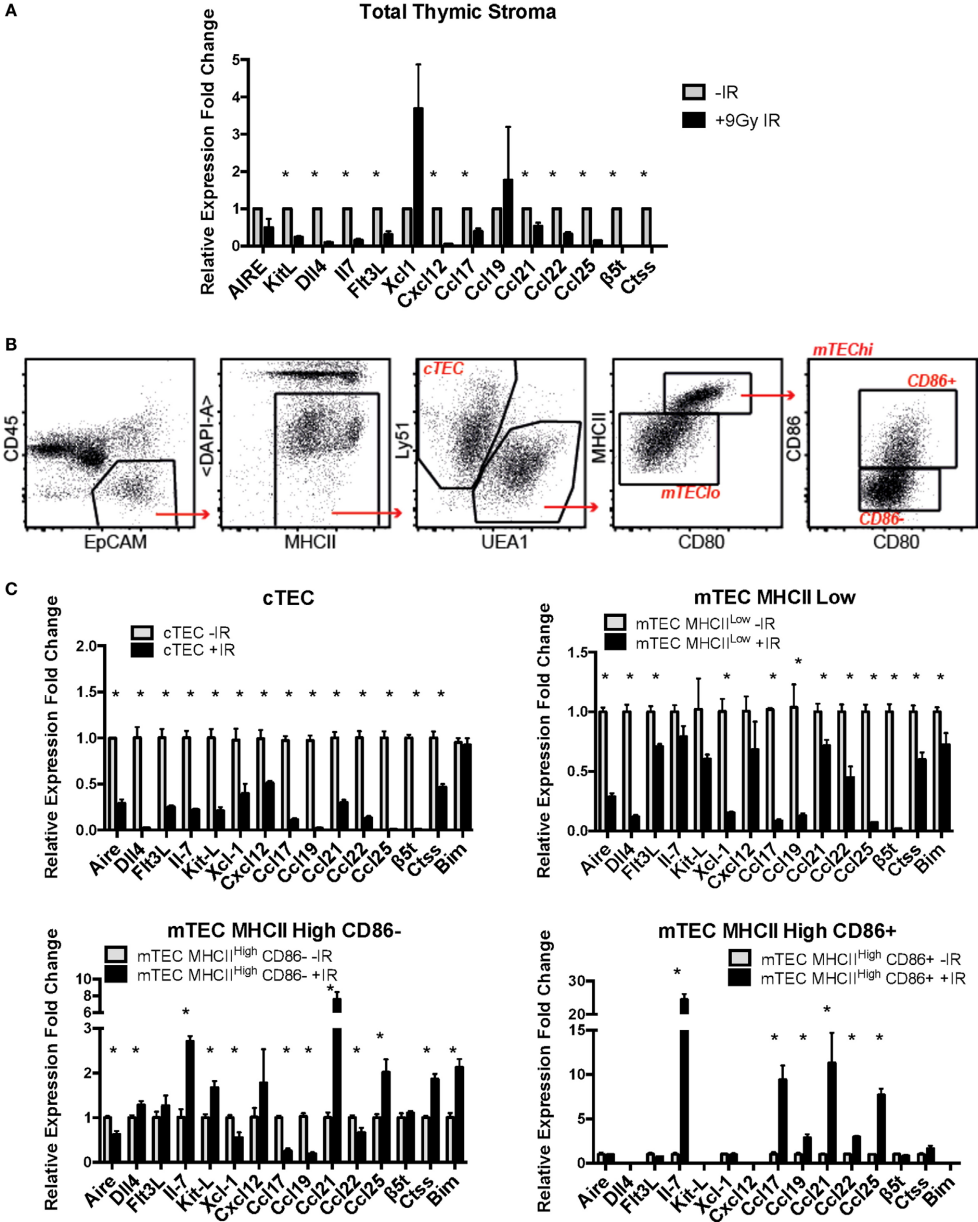
**Effects of ionizing radiation on the functional properties of primary mouse thymic epithelial cell (TEC) subpopulations**. **(A)** mRNA expression levels of TEC functional factors in total primary mouse thymic stroma. All values were normalized against Gapdh and expressed relative to the untreated sample. Graphs show the average of four biological replicates. *p < 0.05, multiple *t*-tests with Holm–Sidak posttest correction. **(B)** Gating strategy for the sorting of mouse primary cTEC, mTEC Low, mTEC High CD86-, and mTEC High CD86^+^ subpopulations. **(C)** mRNA expression levels of TEC functional factors in mouse primary sorted TEC subpopulations. Data were normalized against Gapdh and expressed as fold change relative to the untreated sample. All values correspond to the average of three technical replicates and one biological sample corresponding to 20 thymi per group pooled together prior to the analysis. Error bars represent the SEM. *p < 0.05, multiple *t*-tests with Holm–Sidak posttest correction.

Finally, we investigated the effects of IR treatment on the functional properties of primary mouse TECs. To do so, mRNA expression of a number of genes known to have an important role in TEC function *in vivo* was analyzed with or without IR treatment. Initial experiments were carried out with mRNA isolated from lymphocyte-depleted total thymic stroma. These preparations showed a marked and consistent decrease in expression of most of the genes analyzed, including KitL, Dll4, IL-7, Flt3L, Ccl17, Ccl21, Ccl22, and Ccl25 (Figure 6A), suggesting that the function of the thymic stroma may be compromised following exposure to IR.

In light of these preliminary results, we investigated the expression of these genes in sorted subpopulations of thymic stromal cells from control and irradiated mice (Figures 6B,C; Figures S4 and S5 in Supplementary Material). In addition, given the important role of Bim upregulation in response to IR for survival of mTEC 3–10 cells previously described, mRNA expression of Bim was also included in the analysis. Gene expression in sorted cTEC, mTEC MHCII^Low^, mTEC MHCII^High^, CD86^−^, and mTEC MHCII^High^, CD86^+^ sorted cells (untreated or irradiated) was analyzed by real-time PCR. Due to the low number of mTEC MHC^High^, CD86^+^ obtained from irradiated mice, a cDNA amplification step was necessary in order to obtain enough material for complete analysis. First of all, gene expression of the different TEC functional factors was compared among the different cell types in order to confirm cell identity and establish the relative contribution of each of the cell types to the overall gene expression in the thymus. The different genes analyzed were classified according to the information available from the *Immunological Genome Project (Immgen)* database into genes that are highly expressed in cTECs and progressively lower in the different mTEC subtypes (such as β5t, Il-7, Dll4, KitL, Cxcl12, Ccl21, or Ccl25) and genes that are lowly expressed in cTECs and increase progressively in mTECs (such as Aire, Ctss, Xcl1, Ccl17, Ccl19, or Ccl22). We detected expression of genes traditionally described as mTEC-specific (such as Aire) in cTECs and *vice versa*. However, comparison among cell types confirmed that our gene expression data nicely correlated with the information found in the *Immgen* database and that the expression of mTEC-specific genes in cTECs and cTEC-specific genes in mTECs was extremely low in comparison (Figure S6 in Supplementary Material). Then, IR-induced variation in the expression of TEC functional factors was analyzed. cTECs seemed to be the stromal cell subpopulation most affected by irradiation, showing the most pronounced decrease in all of the studied genes (Figure 6C). mTEC MHCII^Low^ cells also showed a significant decrease in all genes, although to a lesser extent than cTECs. In contrast, both mTEC MHCII^High^ (CD86 positive and negative) subpopulations had a quite similar response to IR treatment, showing downregulation of some genes but also upregulation of others. Among the genes downregulated in mTEC MHCII^High^, CD86^−^ cells were Aire, Xcl-1, Ccl-17, Ccl-19, and Ccl-22, whereas they showed upregulation of Il-7, KitL, Ccl21, and Ccl25. In contrast, mTEC MHCII^High^, CD86^+^ cells showed upregulation in Il-7, Ccl17, Ccl21, and Ccl25 with no change in Aire, Flt3L, or Xcl-1 expression (Figure 6C). Interestingly, all genes upregulated in mTEC MHCII^High^ cells were very weakly expressed in these cells compared to mTEC MHCII^Low^ cells and most especially cTECs. This response pattern probably explains why expression of these genes was downregulated in the total thymic stromal extract analyzed previously (Figure 6A; Figure S6A in Supplementary Material). In line with this, no significant changes were detected in total thymus expression of Aire and Xcl-1, corresponding with the results found for mTEC MHCII^High^, CD86^+^ cells, which are the primary contributors to the expression of these genes (Figure 6A; Figure S6 in Supplementary Material). Interestingly, in the case of Bim, MHCII^High^, CD86^−^ cells showed a significant induction in Bim mRNA expression in response to IR, while mTEC MHCII^Low^ cells showed a mild but significant downregulation, and cTECs showed no changes. Overall, our data suggest that ionizing radiation causes profound changes in expression of many genes encoding factors critical for thymic epithelial function and thymocyte differentiation.

## Discussion

Thymic epithelial cells are one of the main components of the thymic stroma, and they control the homing, proliferation, differentiation, and selection of thymocyte progenitors throughout the process of becoming a mature, functional, and self-tolerant T cell (1, 7). Following total body irradiation (TBI) and BMT, reconstitution of the T cell compartment takes several weeks and requires a fully functional thymus (31). During this period when *de novo* T cell production is impaired and the T cell compartment incapable of mounting specific immune responses, patients are highly susceptible to infectious diseases, disease relapse, and graft-vs.-host disease (32). For this reason, investigating the main causes of poor thymic functionality following BMT is critical to improve the outcomes of this therapy. Surprisingly, there is very little published information available on the functional outcomes of irradiation or other modalities of cytoreductive regimens on thymic stromal cell function. Historically, demonstration that host thymic stroma retained functionality following irradiation came from the seminal papers of Bevan demonstrating the phenomenon of positive selection. Thus, in MHC incompatible radiation bone marrow chimeras, the functional MHC-restricted T cell repertoire of peripheral T cells derived from donor HSC became that of the MHC of the irradiated host and not that of the original bone marrow donor [Bevan (33); Fink and Bevan (34)]. It is now known that cTEC mediate positive selection. Far less attention has been paid to the ability of the post-irradiated thymic stroma, in chimeras, in this case mTEC, to orchestrate negative selection of the T cell repertoire. This involves the re-expression, including the appropriate mRNA splicing, of tissue-specific genes in TECs.

Clinical studies have shown that reduced-intensity cytoreductive regimens result in enhanced T lymphopoiesis (including higher numbers of CD4^+^ T cells, greater T-cell receptor diversity, and higher peripheral T-cell receptor excision circle frequency) (35–37), suggesting that deleterious effects on the thymic stroma are directly linked with the efficiency of the recovery of the T cell compartment. Therefore, development of new strategies to improve T cell production in the thymus requires finding ways to protect the thymic stroma from the insults derived from the BMT process, including DNA damage caused by TBI and chemotherapeutic drugs. To try and understand in some detail the DDR of TEC, we have begun by using continuous growing cell lines representative of cTEC and mTEC, respectively. Some of the results obtained with these cell lines have then been applied to semi-purified preparations of fresh thymic stromal cells. Finally, preliminary experiments are reported on FACS-purified subpopulations of TEC.

We, therefore, began by studying in detail the DDR of two different TEC lines (one cortical and one medullary): cTEC 1–2 and mTEC 3–10. The DDR is the signaling network that allows cells to detect and respond to lesions in their DNA (23) that in physiological conditions follows endogenous damage mediated by free radicals and replicative stress. However, development of this DDR allows cells to respond to damage mediated by external sources such as that caused by ionizing radiation. Although this signaling pathway is present in every cell and is conserved throughout evolution, there is a high variability in the way different cell types respond to insults in their DNA, with different cell types showing distinct DNA repair efficiency and kinetics, repair pathway choice (non-homologous end joining vs. homologous recombination), checkpoint activation or sensitivity to apoptosis, or senescence (38). Comparison between the radio-sensitivity of TEC lines (mTEC 3–10 and cTEC 1–2) and the ST4.5 CD4/CD8 DP T cell line by clonogenic survival assays demonstrated a much higher radio-resistance of the TEC lines than the DP T cells used as radio-sensitive control (Figures 1C,D). When comparing the TEC lines to each other, cell type-specific differences were also observed. While the survival curves were similar for both cell lines at low IR doses (up to 4 Gy), cTEC 1–2 cells showed significantly higher radio-sensitivity at higher doses (Figure S2A in Supplementary Material). In line with this, cleaved Caspase-3 analysis showed a higher propensity of cTEC 1–2 cells to undergo apoptosis in response to both IR and staurosporin treatment (Figure 5) than mTEC 3–10 cells. Cell cycle checkpoint regulation in response to IR also showed a faster recovery of cTEC 1–2 cells from the IR-induced cell cycle arrest, which may be partially explained by their lower expression of checkpoint regulators, such as Cdkn1a (p21) and Cdc25c. Commercial DDR qPCR array analysis also revealed significantly higher expression of approximately 30% of all genes analyzed in mTEC 3–10 cells. These genes mainly encoded DNA repair factors, such as Rad51b, Rad51c, Rad52, Fancd2, Brca1, Brca2, Lig4, or Prkdc (DNA-PKcs), probably indicating a more robust DDR in these cells (Figure 4). The higher presence of DNA-PKcs in mTECs is of special importance since it plays a very important role in their function in T-cell negative selection, acting as a co-factor for Aire-mediated de-repression of tissue-restricted antigen expression (39, 40).

Our group has previously identified hypoxia as an enhancer of the DDR of mesenchymal stromal cells (27). For this reason, we also studied the effects of hypoxia on the radio-resistance of our TEC cell lines. Interestingly, only mTECs showed a cell type-specific responsiveness to hypoxia, which increased their sensitivity to IR. Although growth curves and colony formation assays demonstrated a faster growth rate of mTEC 3–10 cells in hypoxia, clonogenic survival was significantly lower in this condition (Figure 1). No difference was observed in checkpoint regulation or DNA repair capacity of mTEC 3–10 cells cultured at different oxygen tensions (Figures 2 and 3). However, cleaved Caspase-3 analysis showed higher apoptosis rates in hypoxic mTEC 3–10 cells in response to treatment with both IR and staurosporin (Figures 5A,B). In order to study the mechanism behind this phenotype, a detailed analysis of pro- and anti-apoptotic protein levels was performed, evidencing a stronger induction of Bim expression in hypoxia, compared to normoxia. Bim is a very strong apoptosis inducer thanks to its ability to bind to many anti-apoptotic proteins (Mcl-1, Bcl-2, Bcl-xL, Bcl-w, Bfl-1, and Epstein–Barr virus BHRF-1) as well as directly binding to the pro-apoptotic proteins Bax and Bak and directing them to the mitochondrial membrane and inducing its permeabilization (41). Since this pro-apoptotic protein increase was not accompanied by any specific anti-apoptotic protein induction that could counteract the effects of Bim, it is likely that this is one of the main drivers of the higher susceptibility to undergo apoptosis of hypoxic mTEC 3–10 cells. In light of these results, Bim mRNA expression changes in response to IR were subsequently studied in primary sorted TEC subpopulations (Figure 6C), demonstrating that mTEC 3–10 cells behave similarly to mTEC MHCII^High^ CD86^−^ cells, which show an induction in Bim expression in response to IR. In contrast, primary cTECs do not show any significant induction of Bim mRNA expression, in line with the very modest Bim protein upregulation observed in cTEC 1–2 cells.

Our preliminary data with whole thymic stroma preparations showed a marked decrease in the mRNA levels of most of the transcripts analyzed (Figure 6A). Previous studies have shown depletion of mTEC and cTEC populations and enrichment of fibroblastic components in the thymi of irradiated mice (42). Other authors have described similar decreases of specific transcripts, such as IL-7 or Ccl25, although these changes have been mainly attributed to changes in thymic cellularity (43, 44). For this reason, we performed a more detailed analysis of the gene expression of purified sorted TEC types, in order to exclude the possibility that the decrease in mRNA levels was due to a decrease in total TEC numbers and not to a specific downregulation of gene expression. In contrast to the results mentioned above, our experiments did not show differences in the number of sorted cells between irradiated and un-irradiated groups (data not shown), although this is probably due to the fact that our sorts were performed 24 h after irradiation whereas other groups have studied changes in TEC numbers at longer time points after IR (42, 44). Our analysis of different purified TEC subpopulations individually confirmed the overall functional factor downregulation and revealed cTECs as the most affected by ionizing radiation (Figure 6C). These molecules have important roles in attraction, commitment, survival, proliferation, migration, and selection of thymocytes throughout their development (1, 7). Previous work by different groups has shown the important implications of this decrease of TEC functional factors in T-cell reconstitution following BMT. Observations by Zlotoff et al. and Zhang et al. revealed a marked decrease in thymic seeding by progenitors in irradiated thymuses in comparison to un-irradiated ones (43, 45), which could be rescued by supplementation with Ccl21 and Ccl25 (43). Other studies have also shown enhanced posttransplantation thymic recovery by exogenous administration of IL-7 (46, 47) or Flt3l (48, 49). Thus, elucidation of the mechanisms behind damage-induced loss of thymic function may be useful for the design of promising strategies to improve T-lineage recovery following BMT.

In conclusion, to the best of our knowledge, we have for the first time studied in detail the DDR of TECs and the short-term effects of ionizing radiation on their expression of many genes that are essential for T cell development. We have shown that TECs exhibit a relatively high radio-resistance, although IR has detrimental effects in their survival and functionality, inducing a profound downregulation of functional factors in primary murine TECs. We have also shown how cTECs and mTECs respond differently to DNA damage, by displaying differential checkpoint recovery and sensitivity to undergo apoptosis in response to IR, as well as differential expression of DDR genes such as DNA repair factors or proteins involved in cell cycle regulation. Finally, we have demonstrated that hypoxia reduces the radio-resistance of our mTEC 3–10 cell line trough the upregulation of the pro-apoptotic protein Bim. These findings constitute a first step toward understanding TEC response to IR and the mechanisms behind their radio-resistance, which is crucial for improving the outcomes of BMT and promoting successful T cell reconstitution.

## Author Contributions

IC-A: conception and design, collection and assembly of data, data analysis and interpretation, and manuscript writing; TB: collection and assembly of data and manuscript correction; LM: collection and assembly of data; NL: conception and design, data analysis and interpretation, manuscript writing, and final approval of manuscript; RC: conception and design, financial support, data analysis and interpretation, manuscript writing, and final approval of manuscript.

## Conflict of Interest Statement

The authors declare that the research was conducted in the absence of any commercial or financial relationships that could be construed as a potential conflict of interest.
