# Supplementary material for: Differential Response of Mouse Thymic Epithelial Cell Types to Ionizing Radiation-Induced DNA Damage
Source: Front Immunol. 2017 Apr 13;8:418. doi: 10.3389/fimmu.2017.00418 (PMC5389985; doi:10.3389/fimmu.2017.00418)
Supplement: Supplementary file 1 [file Data_Sheet_1.DOCX]

Supplementary Material

**Differential response of mouse thymic epithelial cell types to ionizing radiation-induced DNA damage**

Irene Calvo-Asensio^1,2^, Thomas Barthlott^3^, Lilly von Muenchow^4^, Noel F. Lowndes^2^, Rhodri Ceredig^1*^

^1^Regenerative Medicine Institute, Department of Physiology, National Centre of Biomedical Engineering Science and School of Medicine, Nursing and Health Sciences, National University of Ireland, Galway, Ireland;

^2^Genome Stability Laboratory, Centre for Chromosome Biology, School of Natural Sciences, National University of Ireland, Galway, Ireland;

^3^Laboratory of Pediatric Immunology, Center for Biomedicine, Department of Clinical-Biological Sciences, University of Basel and The University Children's Hospital (UKBB), Basel, Switzerland;

^4^Developmental and Molecular Immunology, Department of Biomedicine, University of Basel, Basel, Switzerland

*** Correspondence:** Rhodri Ceredig: rhodri.ceredig@nuigalway.ie

**1. Supplementary Methods**

**1.1. Isolation and Sorting of Mouse Thymic Epithelial Cell Subpopulations**

For the TEC isolation, thymic lobes were separated, cleaned from fat and connective tissue, pierced with watchmaker forceps and placed in PBS containing Liberase TM research grade (1 Wünsch unit/ml; Roche) at 37°C. After 10 min., lobes were gently resuspended using a 1 ml pipet tip cut off to provide a 3-4 mm opening. Lobes were further incubated for 5 min. intervals at 37°C and resuspended using increasingly smaller tip openings until a homogeneous cell suspension was achieved. Cells were washed with IMDM containing 10% FCS and 5 mM EDTA, resuspended in PBS/2% FCS at a concentration of 100 x 10^6^/ ml and stained with biotinylated EpCAM for 15 min. on ice. Washed cells were resuspended at 250 x 106/ ml and 60μl/ml anti Biotin beads (Miltenyi) were added for 15 min. at 4°C. Washed cell preparations were then separated on the AutoMACSpro (Miltenyi) with the POSSEL program. The enriched TEC fraction was re-stained with EpCAM PE-Cy7 and Streptavidin PE-Cy7, CD45 Alexa700, Ly51 PE, UEA-1 FITC, MHCII APC-Cy7, CD80 PerCP-Cy5.5 and CD86 APC. Dead cells were discriminated by DAPI and TEC subsets were sorted on a FACSAria (BD Biosciences).

**1.2. Flow cytometry**

1.2.1. Surface marker analysis by surface staining

Cells were trypsinized to obtain a single cell suspension and counted. 5x10^5^ cells per sample were resuspended in FACS buffer (2% FCS, 0.05% Sodium Azide, PBS) and stained with the appropriate primary antibodies or isotype controls. If applicable, cells were then washed and stained with fluorescently-labeled streptavidin prior to analysis using a BD Accuri^TM^ C6 flow cytometer (BD Biosciences) and FlowJo® software (TreeStar Inc., OR, USA).

1.2.2. Cell cycle analysis using BrdU–Propidium Iodide Staining

Cells were labeled for 45 min with 25 μM 5’‐bromo‐deoxyuridine (BrdU) (Sigma Aldrich), washed in PBS and re‐suspended in growth medium. Cells were harvested at the indicated time points post irradiation (3 Gy), fixed in ice‐cold 70% ethanol and stained with anti‐BrdU and FITC-conjugated anti‐mouse IgG antibodies and Propidium Iodide (PI) / RNase staining buffer (BD Biosciences) as previously described (26). The progression of cells through the cell cycle was analyzed by measuring the percentage of BrdU-positive cells in each G1 phase until 24 h post IR using a BD FACS Canto® flow cytometer (BD Biosciences) and FlowJo® software (TreeStar Inc., OR, USA).

1.2.3. G2/M checkpoint analysis using phospho‐Histone H3 Ser10‐Propidium Iodide Staining

Cells were harvested at the indicated time‐points post irradiation (3 Gy), washed in PBS and fixed in ice‐cold 70% ethanol. Following permeabilization in PBS / 0.25% Triton X‐100, cells were sequentially stained with anti‐phospho‐histone H3 (Ser10) and FITC‐conjugated anti‐rabbit IgG antibodies for 2 hrs and 30 mins at room temperature, respectively, separated by two washing steps in PBS / 1% BSA. Cells were then re‐suspended in PI / RNase staining buffer (BD Biosciences) and mitotic index analyzed up to 24 hrs post irradiation using a BD FACS Canto® flow cytometer (BD Biosciences) and FlowJo® software (TreeStar Inc., OR, USA).

1.2.4. Analysis of Apoptosis using cleaved Caspase-3 Staining

TEC cell lines were harvested at the indicated time‐points post irradiation (3 Gy), washed in PBS and fixed with 2% PFA. Cells were then permeabilized using PBS / 0.5% Saponin and sequentially stained with anti-Caspase-3 and FITC-conjugated anti-rabbit IgG antibodies diluted in PBS / 1% BSA for 2h and 1h respectively at 37ºC, separated by 2 washes with PBS / 1% BSA. Apoptotic cells were quantified by flow cytometry as Caspase-3 positive cells up to 96h post IR using a BD Accuri^TM^ C6 flow cytometer (BD Biosciences) and FlowJo® software (TreeStar Inc., OR, USA). Staurosporine-treated cells were used as positive control for this assay.

**1.3. Antibodies**

For western blotting, anti-phospho-Histone H2A.X (Ser139) mouse monoclonal antibody (Millipore), anti-H2AX rabbit polyclonal antibody, anti-ATM [2C1(1A1)] mouse monoclonal antibody, anti-DNA-PKcs (Y393) rabbit monoclonal antibody, anti-DNA Ligase IV rabbit polyclonal antibody, anti-Rad51 rabbit polyclonal antibody (Abcam), anti-ATR (N-19) goat polyclonal antibody, anti-Chk2 (H-300) rabbit polyclonal antibody, anti-Noxa (FL-103) rabbit polyclonal antibody (Santa Cruz Biotechnology Inc.), anti-p53 1C12 mouse monoclonal antibody, anti-Bcl-XL (54H6) rabbit monoclonal antibody, anti-Bcl-2 (D17C4) rabbit monoclonal antibody, anti-Bim (C34C5) rabbit monoclonal antibody, anti-Puma (E1S7A) rabbit polyclonal antibody, anti Mcl-1 (D35A5) Rabbit monoclonal antibody, anti-Bak (D2D3) rabbit monoclonal antibody, anti-Bax rabbit polyclonal antibody, anti-pBad (S112) (7E11) mouse monoclonal antibody, anti-Bad rabbit polyclonal antibody (Cell Signaling Technologies), anti-Xiap (2F1) mouse monoclonal antibody (Enzo Life Sciences), anti-β-Actin rabbit polyclonal antibody (Sigma-Aldrich), Pierce horseradish peroxidase (HRP)-conjugated rabbit anti-mouse IgG antibody, Immuno-Pure HRP-conjugated goat anti-rabbit IgG antibody (Thermo Scientific), IRDye® Goat anti-Mouse and Goat anti-Rabbit IgG (LiCor) were used. For immunofluorescence staining, anti-phospho-Histone H2A.X (Ser139) mouse monoclonal antibody (Millipore) and anti-Rad51 rabbit polyclonal antibody (Abcam) were used. Secondary fluorescein (FITC)-conjugated AffiniPure F(ab0)2 Fragment goat anti-mouse IgG antibody and Texas Red-conjugated AffiniPure F(ab0)2 Fragment goat anti-rabbit IgG antibody (Jackson ImmunoResearch Laboratories Inc.) were used. For flow cytometry, anti-50-bromodeoxyuridine (BrdU) mouse monoclonal antibody (BD Biosciences), anti-Caspase 3 rabbit polyclonal antibody (Abcam), anti-phospho‐Histone H3 (Ser10) rabbit polyclonal antibody (Millipore), FITC-conjugated anti-rabbit IgG (whole molecule) (Bethyl) FITC-conjugated anti-mouse IgG (whole molecule) antibody (Sigma-Aldrich), FITC-anti-CD45 (30-F1), FITC Rat IgG2b, κ Isotype Control (A95-1), FITC-Anti-Ly51 (6C3), FITC Rat IgG2a, κ Isotype Control (R35-95), APC-anti-IAb (AF6-120.1), APC Mouse IgG2a κ Isotype Control  (G155-178), FITC-anti-EPCAM (G8.8) (BD Biosciences), FITC-anti-IAb (AF6-120.1), PE-anti-Ly51 (6C3), PECy7-anti-CD45 (30-F11), APC-Cy7-anti-I-A/E (M5/114.15), Streptavidin-labelled PerCP-Cy5.5 (BioLegend), biotinylated anti-EpCAM (G8.8) (DSHB, University of Iowa), Cy5-UEA-1 and biotynilated UEA-1 lectin (Vector Laboratories) were used.

# Supplementary Figures and Tables

For more information on Supplementary Material and for details on the different file types accepted, please see [here](http://home.frontiersin.org/about/author-guidelines#SupplementaryMaterial).

## Supplementary Figures

**
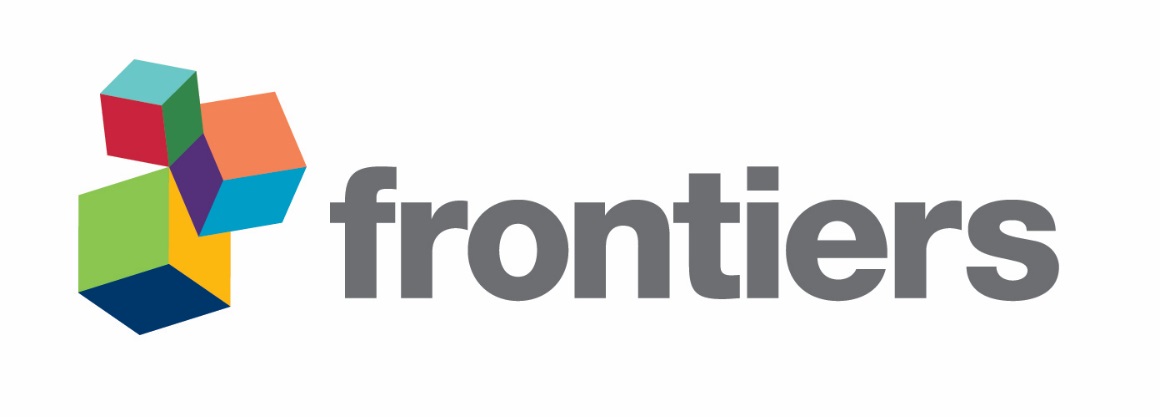
**

**Supplementary Figure S1**: *Thymic epithelial cell line characterization*. **(A)** Morphological characteristics of mTEC 3-10 (left) and cTEC 1-2 cell lines. Scale bar corresponds to 125μm. **(B)** Flow cytometry analysis of surface marker expression of mTEC 3-10 and cTEC 1-2 cell lines. Isotype control is indicated in light blue while surface markers are indicated in dark blue. **(C)** MFI of isotype control and surface marker staining of mTEC 3-10 and cTEC 1-2 cell lines. Average of 3 replicates is shown, with error bars indicating the standard error of the mean (SEM). * p<0.05, multiple t-tests with Holm-Sidak post correction.

**Supplementary Figure S2**: *Thymic epithelial cell line DNA damage response*. **(A)** Clonogenic survival of normoxic mTEC 3-10, cTEC 1-2 and ST4.5 cell lines. **(B)** Cytograms of mTEC 3-10 and cTEC 1-2 cells stained for BrdU incorporation and DNA content (propidium iodide) in 21% or 5% O_2_ at different time points following BrdU pulse, with or without treatment with 3Gy of IR. Representative gating strategy for the identification of BrdU+ G1 cells is shown in black, while gates for the identification of cells in different phases of the cell cycle are shown in green (G1 cells), blue (S-phase cells) and red (G2/M cells).

**Supplementary Figure S3***: Double strand break repairby HR kinetics in TECs.* **(A)** Representative images of mTEC 3-10 and cTEC 1-2 nuclei stained for Rad51 IRIF, in 21% or 5% O 2 , 0-24h post 3Gy irradiation. **(B)** Average number of Rad51 IRIF per nucleus 0-24h post-IR.

**Supplementary Figure S4**: *Cell sorting strategy of mouse primary TEC subpopulations.* Gating strategy and post-sort purity data of untreated TEC subpopulations.

**Supplementary Figure S5**: *Cell sorting strategy of mouse primary TEC subpopulations.* Gating strategy and post-sort purity data of irradiated TEC subpopulations.

**Supplementary Figure S6**: *Mouse primary sorted TEC subpopulation gene expression comparison.* mRNA expression of TEC functional factors in untreated TEC subpopulations, expressed as 2^(-ΔCt) normalized to Gapdh expression. Genes are classified in two groups according to their expression pattern across TEC subpopulations: **(A)** genes expressed highly in cTECs and with decreasing expression across the mTEC subpopulations and **(B)** genes expressed lowly in cTECs and with increasing expression across the mTEC subpopulations. All values correspond to the average of three technical replicates and one biological sample corresponding to twenty thymi pooled together prior to the analysis. * *p<0.05*, multiple t-tests with Holm-Sidak post-test correction.
