# Supplementary figures and images for: Differential Response of Mouse Thymic Epithelial Cell Types to Ionizing Radiation-Induced DNA Damage
Source: Front Immunol. 2017 Apr 13;8:418. doi: 10.3389/fimmu.2017.00418 (PMC5389985; doi:10.3389/fimmu.2017.00418)

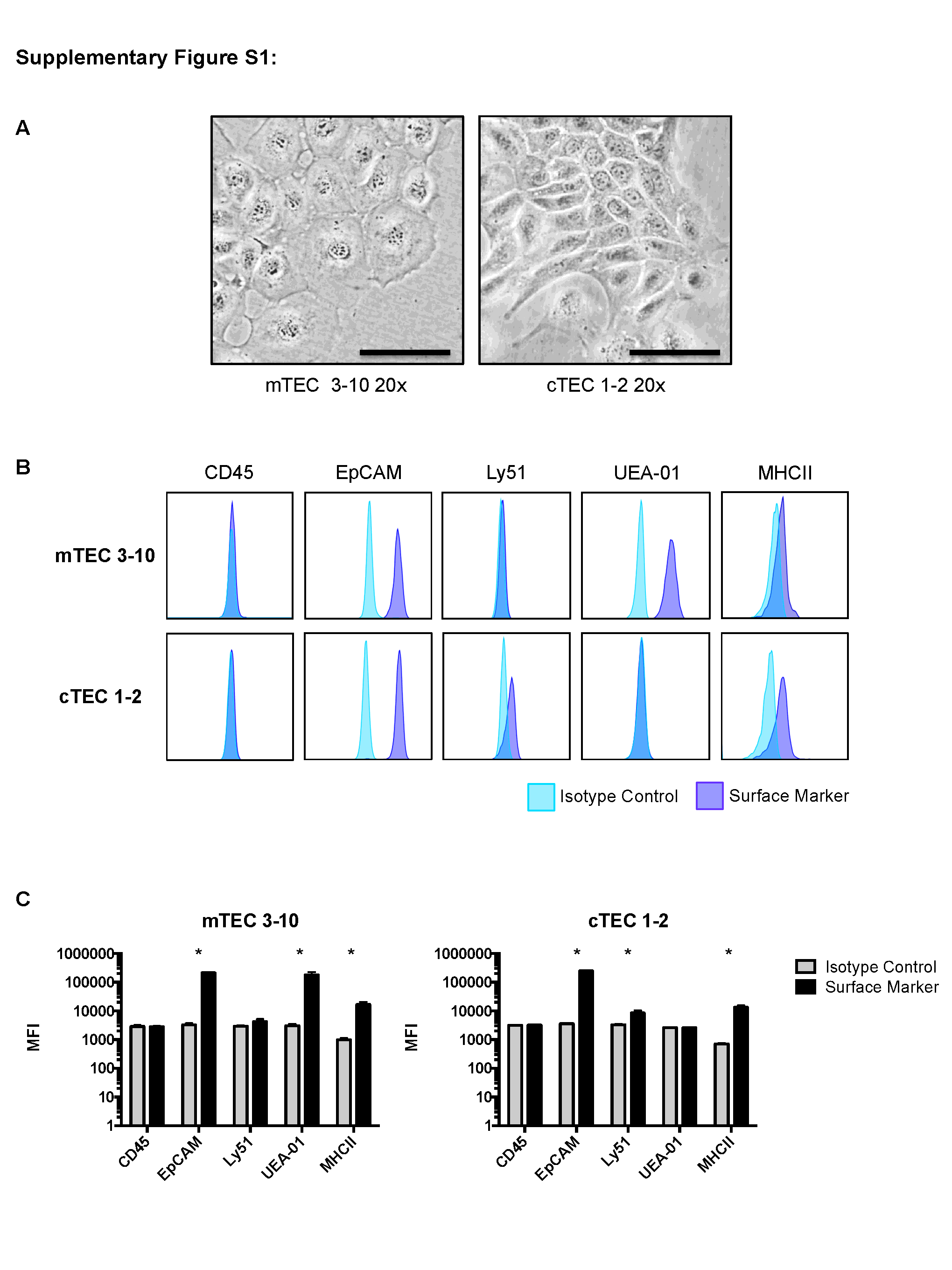

Supplement: Supplementary file 2 [file Image_1.TIF]

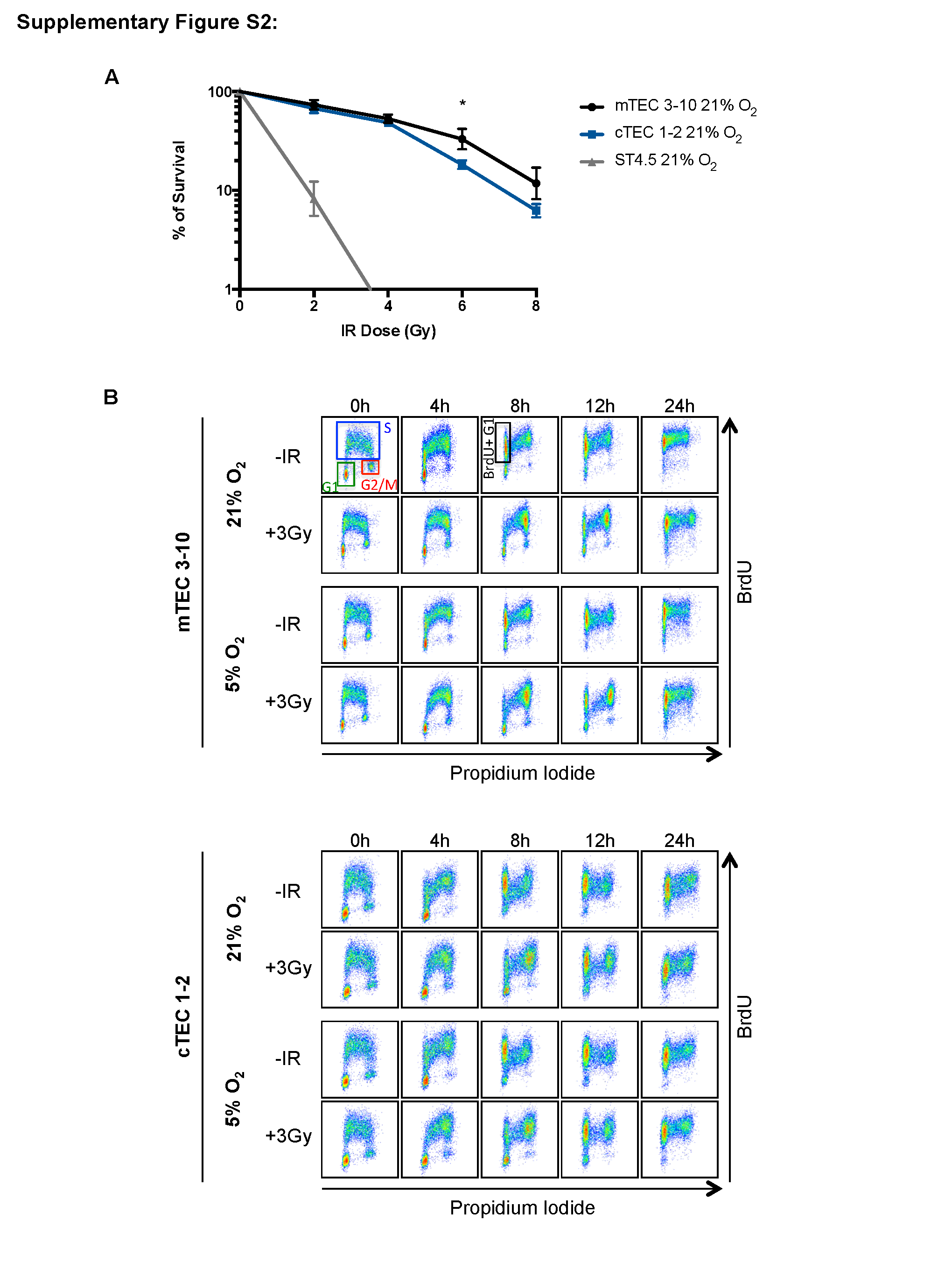

Supplement: Supplementary file 3 [file Image_2.TIF]

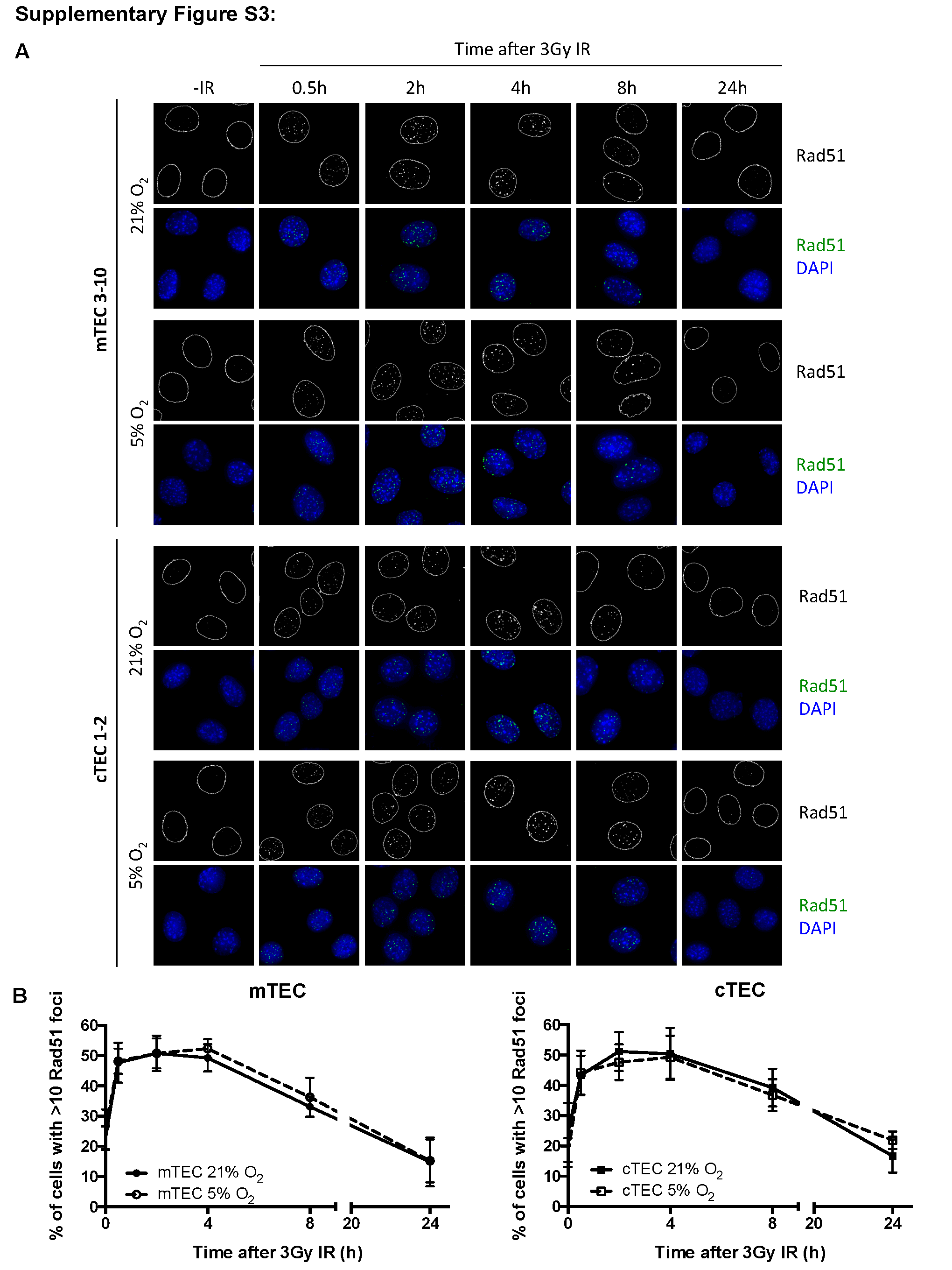

Supplement: Supplementary file 4 [file Image_3.TIF]

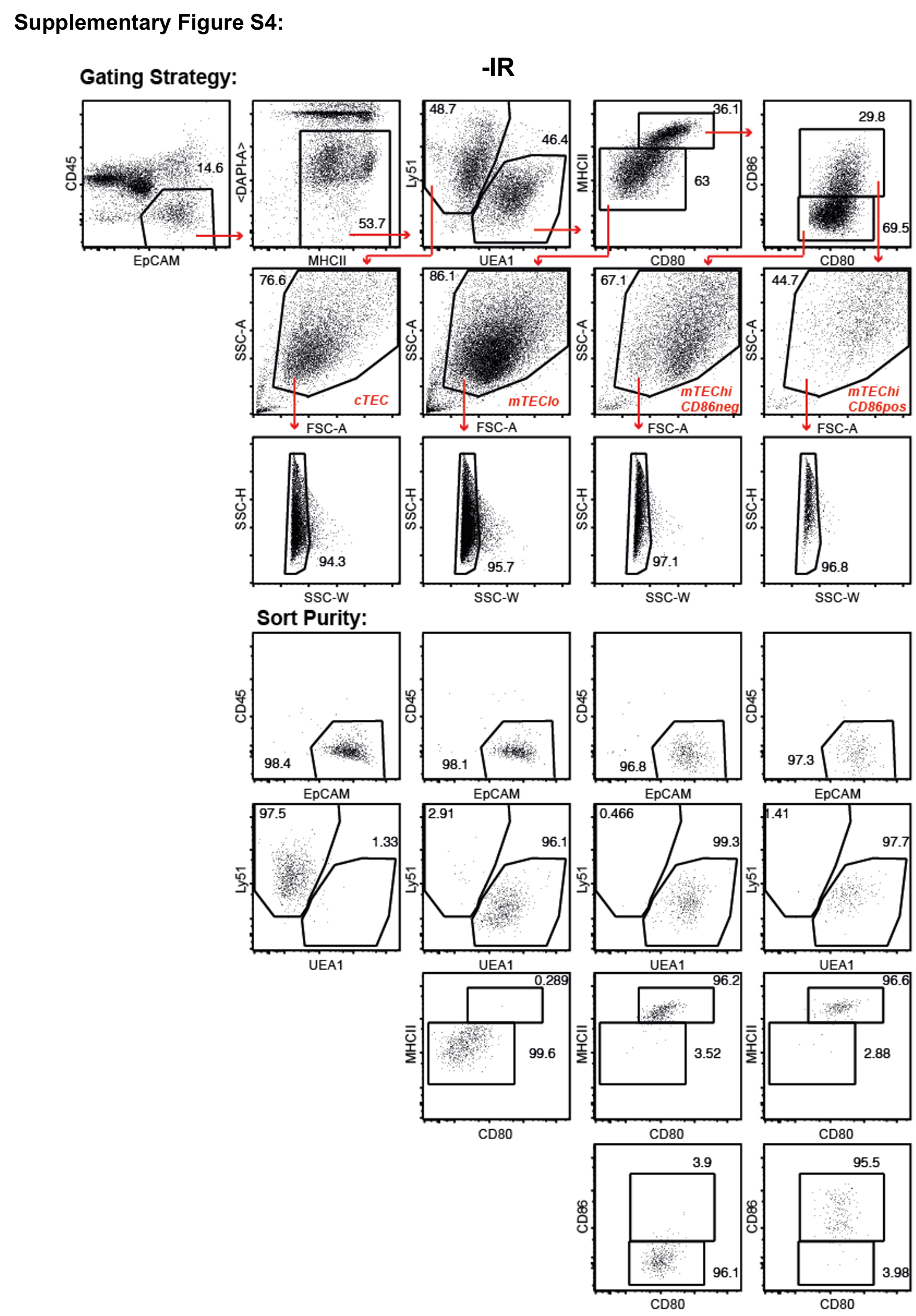

Supplement: Supplementary file 5 [file Image_4.TIF]

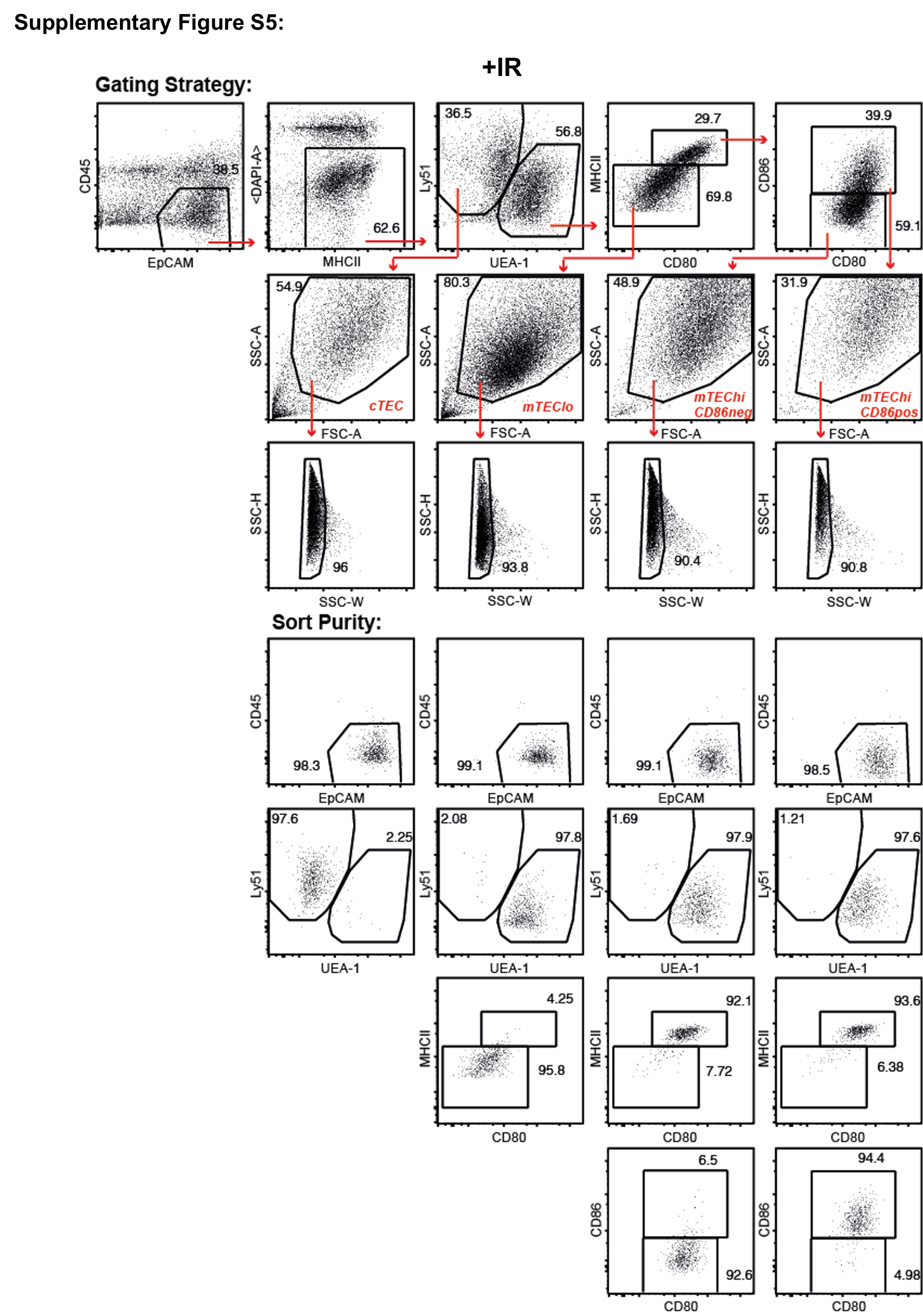

Supplement: Supplementary file 6 [file Image_5.TIF]

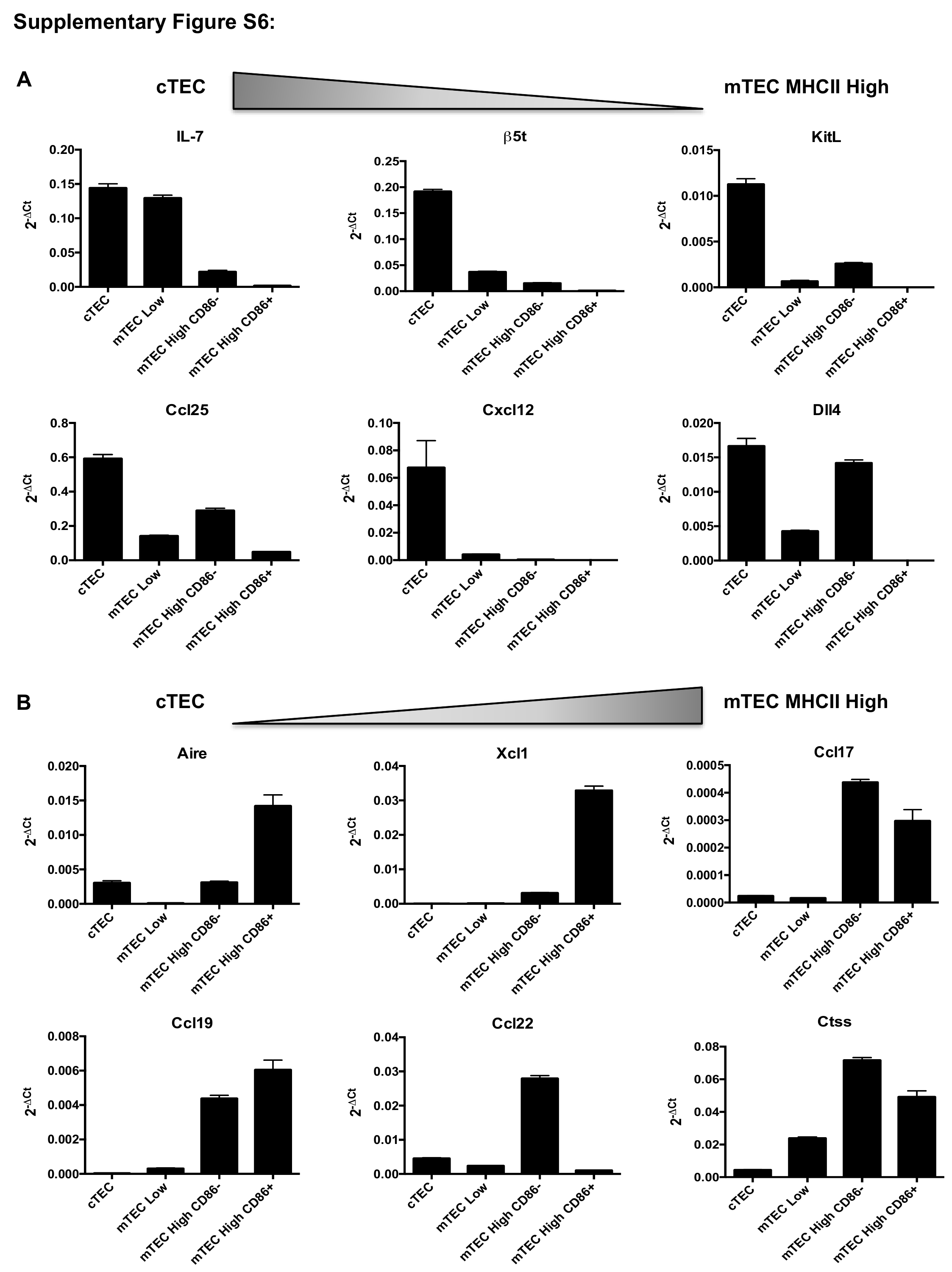

Supplement: Supplementary file 7 [file Image_6.TIF]
